# Supplementary material for: Are carbon emissions trading and green financial instruments synergistic? -Comprehensive quantitative research based on content analysis
Source: PLoS One. 2024 Mar 7;19(3):e0298601. doi: 10.1371/journal.pone.0298601 (PMC10919601; doi:10.1371/journal.pone.0298601)
Supplement: S1 Data — (ZIP) [file pone.0298601.s001.zip › Data/地级市Z_F工作报告.zip!/║╙─╧/╞╜╢Ñ╔╜╩╨/└┤╫╘╣1⁄2╓┌║┼í╛┬φ┐╦╩2╛▌═°í┐_2016.docx]

各位代表:

现在，我代表市人民政府，向大会报告工作，请予审议，并请各位政协委员和其他列席人士提出意见。

一、“十二五”主要成就及“十三五”奋斗目标

过去五年，面对国内外严峻复杂形势和一系列重大风险挑战，在省委、省政府和市委的正确领导下，全市上下全面落实党的十八大和十八届三中、四中、五中全会精神，深入贯彻习近平总书记系列重要讲话精神，扎实开展党的群众路线教育实践活动，主动适应引领经济发展新常态，紧紧围绕转型发展主题，真抓实干、开拓创新，持续推进转型提速、发展提质、环境提优、幸福提升，基本完成了“十二五”规划主要目标任务。

——综合实力稳步提升。2015年生产总值1335亿元，五年平均增长7.1%(不含汝州市)；人均生产总值33104元，年均增长6.2%。规模以上工业增加值559.7亿元，年均增长9.1%。一般公共预算收入97.2亿元、支出214亿元，分别是2010年的1.36倍和1.66倍。全社会固定资产投资累计完成5210亿元，是“十一五”时期的2.6倍。社会消费品零售总额572亿元，是2010年的1.95倍。城乡居民人均可支配收入分别达到25592元和10450元，是2010年的1.61倍和1.87倍。金融机构存款余额1793.2亿元，是2010年的1.73倍。粮食总产163.9万吨，比2010年增加11.2万吨。

——结构调整成效明显。非煤产业比重提升15.5个百分点，高新技术产业和装备制造业比重分别提升9.8个和7.8个百分点，七大高耗能行业比重下降14.3个百分点。产业集聚区规模以上工业增加值占全市的49.2%，提升18.4个百分点，市高新技术开发区晋升为国家级高新区，郏县产业聚集区创建为省级经济技术开发区，宝丰高新技术开发区晋升为省级高新区。服务业“两区”建设稳步推进，文化旅游、现代物流、金融保险等现代服务业快速发展，尧山温泉旅游度假区入选首批国家级旅游度假区，尧山——中原大佛景区创建为国家5A级景区，6家景区创建为4A级景区，14家金融机构入驻我市，平顶山银行实现跨区域经营，第三产业比重提高11.4个百分点。新建高标准粮田62万亩，培育循环畜牧企业101家，创建为省生态循环畜牧业示范市。科技创新能力持续增强，获国家科技进步奖10项、省科技进步奖53项，新增院士工作站3家、博士后研发基地2家、国家高新技术企业46家、省工程技术研究中心36家，创建为全国科技进步先进市、国家知识产权试点市。节能减排得到加强，万元生产总值能耗下降25.7%。

——城乡面貌深刻变化。成功创建为国家卫生城市、国家森林城市、省文明城市，城镇化率达到51%，提高7.4个百分点。城乡一体化示范区总体规划获批，完成投资274亿元，形成“五横十二纵”道路网络，建成区面积拓展到31平方公里，平顶山博物馆、城市规划展览馆、市民广场等项目建成投用。老城区城中村开发和旧城改造主体完工1106.6万平方米，新建改扩建道路102.4公里，延伸市政管网622.6公里，更新公交车560辆。湛河综合治理征迁安置、截污治污主干工程基本结束，支沟治理和景观绿化加快推进。湛南新城建设取得新进展。集中整治违法占地3073亩，拆除违章建筑222.8万平方米。市数字化城管投入运行，成为全国数字城市建设示范市。郑万铁路在我市先期开工，三洋铁路郏县段开通运行，平宝、宝石、平郏快速通道建成通车。南水北调平顶山段如期通水，移民安置工作受到省政府表彰。新改建国省干线公路636公里、农村公路2530公里，实现了县县通高速、村村通公路。启动建设新农村293个，建设新民居5.1万套，26.5万人脱贫，解决120.1万农村居民和19.3万农村师生饮水安全，农村生活条件明显改善。特别是2014年战胜了建市以来最严重旱情，有力保障了生产生活用水。

——改革开放持续深化。行政体制改革稳步推进，新一轮政府机构改革顺利完成，理顺了产业聚集区、新城区、高新区、石龙区管理体制。非行政许可审批全面取消。在全省率先实行模拟审批，被列为省试点。商事制度改革全面展开，新登记企业数量快速增长。完成27家国有企业改制、22家煤炭企业兼并重组、97座小煤矿关闭，平高集团与国家电网实施战略重组，平煤神马集团被列入省混合所有制经济和市场化选聘职业经理人“双试点”。投融资体制改革步伐加快，7家公司成功上市。城市管理体制改革取得新进展，社会保障制度改革不断深化，医药卫生体制改革加快推进。成功举办第四届、第五届“华合论坛”等重大招商活动，与墨西哥圣路易斯、巴西坎布里乌建立友好城市关系，与德国劳恩堡市签订了友好城市关系协议。2015年实际到位省外资金412.5亿元、利用外商直接投资3亿美元，年均分别增长22.9%和14.3%。进出口总额累计达到26.9亿美元，是“十一五”的1.58倍。

——人民生活不断改善。民生支出占财政支出70%以上。城镇新增就业50.3万人，农村劳动力转移就业29.5万人。企业退休人员养老金连年提高，城镇居民医保、新农合筹资标准和补助水平持续提高，城乡居民养老金、城乡低保标准得到提高，在全省率先建立社会临时救助制度。建成保障性住房8.9万套，实施农村危房改造2.8万户，为历史遗留问题房屋办证1.5万本。新建改建幼儿园277所、中小学402所，城区幼儿入园难和中小学大班额问题有效缓解。普通高考录取率逐年提高。职教攻坚计划顺利完成。河南城建学院、平顶山学院通过本科教学评估。成功举办第二届中国曲剧艺术节、第八届中国杂技金菊奖等大型文化活动，被命名为中国书法城、中国观音文化之乡、中国汝窑陶瓷艺术之乡、中国唐钧基地。完成体育村升级改造。医疗卫生设施得到加强，公共医疗卫生服务体系更加健全。人口计生工作保持全国先进。荣获全国无障碍建设先进城市、全国残疾人工作示范城市，双拥模范城实现“四连冠”。社会治安和安全生产形势总体稳定，食品药品安全防线不断加固，社会更加和谐安定，人民群众幸福感明显提升。

各位代表!

经过五年努力，我市经济社会发展站在新的历史起点上，为全面建成小康社会打下了坚实基础。这是省委、省政府和市委坚强领导的结果，是市人大、市政协大力支持的结果，是全市人民团结奋斗的结果。在此，我代表市人民政府，向广大工人、农民、知识分子、干部，向驻平解放军指战员、武警官兵、公安民警及社会各界人士，致以崇高敬意!向关心支持平顶山发展的海内外友好人士，表示衷心感谢!

回顾“十二五”工作，我们深刻体会到，加快平顶山发展，必须把牢政治方向、强化大局意识，凝聚同心同德、共谋发展的强大合力；必须突出问题导向、抓住主要矛盾，扎实推进转型提速、发展提质、环境提优、幸福提升；必须深化改革开放、提升创新能力，为加速全面转型挖掘潜力、激发活力、增强动力；必须坚持以人为本、秉持民生导向，让发展成果更多惠及全市人民；必须加强党的建设，优化政治生态，为改革发展稳定提供根本保证。这些经验是全市人民智慧的结晶，必须倍加珍惜、发扬光大!

各位代表!

“十三五”时期是全面建成小康社会的决胜阶段，也是我市爬坡过坎、转型攻坚的关键时期。根据市委八届十一次全会关于制定“十三五”规划的建议，市政府编制了《平顶山市国民经济和社会发展第十三个五年规划纲要(草案)》，已印发会议，提请审议。

“十三五”时期经济社会发展的指导思想是：高举中国特色社会主义伟大旗帜，全面贯彻落实党的十八大和十八届三中、四中、五中全会精神，以马克思列宁主义、毛泽东思想、邓小平理论、“三个代表”重要思想、科学发展观为指导，深入贯彻习近平总书记系列重要讲话精神，按照“五位一体”总体布局和“四个全面”战略布局，坚持发展第一要务，积极践行创新、协调、绿色、开放、共享发展理念，以提高发展质量效益为中心，以转型发展为主题，以结构调整为主线，以改革创新为动力，以项目建设为抓手，加快形成引领经济发展新常态的体制机制和发展方式，统筹推进“四化”同步发展，深入推进资源型城市可持续发展示范区建设，着力优化经济结构、转换发展动力，着力保障改善民生、维护和谐稳定，确保如期全面建成小康社会。

奋斗目标是：经济保持中高速增长，地区生产总值年均增长7.5%左右，生产总值和城乡居民人均收入比2010年翻一番以上，转型升级实现突破，人民生活水平和质量普遍提高，改革开放纵深推进，社会建设显著进步，生态环境总体改善，全民素质和社会文明程度明显提高，到2020年全面建成小康社会，力争创建成为全国文明城市，资源型城市转型发展取得质的突破。

实现上述目标，必须坚持创新发展，培育转型新动力；坚持协调发展，增强转型新优势；坚持绿色发展，提升转型新水平；坚持开放发展，拓展转型新空间；坚持共享发展，普惠转型新成果。

各位代表!

展望“十三五”，宏伟蓝图鼓舞人心、催人奋进。我们坚信，经过五年奋斗，我市转型发展一定能够取得重大进展，全面建成小康社会目标一定能够实现，平顶山的明天一定会更加美好!

二、2015年工作回顾

2015年是“十二五”收官之年。一年来，按照省委、省政府和市委决策部署，我们统筹稳增长、调结构、促改革、防风险、惠民生，深入开展“三严三实”专题教育和“三查三保”活动，转中求进、攻坚克难，经济社会发展呈现中速增长、结构优化、民生改善、大局稳定的态势。初步核算，全年生产总值增长6.1%，固定资产投资增长9.2%，社会消费品零售总额增长12.1%，城乡居民人均可支配收入分别实际增长5.8%和7.3%。一般公共预算收入完成1178758万元(含汝州市)，实现了经各级人大常委会调整后的预算目标。我们重点抓了以下工作：

(一)全力稳增长保态势，经济运行保持平稳。坚持把稳增长保态势作为全局工作的突出任务，着力稳定经济运行。加强调控稳增长。出台了稳增长保态势若干专项方案、促进房地产市场平稳健康发展的意见等政策措施，生产总值增速逐季回升，在全省位次前移3位。深化服务稳增长。深入开展企业大服务专项行动，高度关注传统行业中的困难企业，定期召开与重点企业联席会议，现场办公、解决问题。设立企业还贷周转金、中小企业增信基金和新兴产业创投基金，其中还贷周转金累计使用120次、周转额13.3亿元。报批各类建设用地1.4万亩，完成土地收储4088亩。对各县(市、区)分类指导、加压督促，帮助稳定经济。扩大投资稳增长。突出抓好“五个一批”项目建设，完善模拟审批和项目推进机制，每季度组织一次集中开工活动，孟平铁路复线等工程进展顺利。争取到中央资金8.3亿元、国家专项建设基金18.9亿元。重点项目建设完成投资630亿元。优化环境稳增长。以“一征集、三查(察)、两移交、一曝光”为基本方法，强力推进懒政怠政、为官不为问题专项治理，发展环境进一步优化。

（二）全力调结构转方式，转型升级步伐加快。产业结构持续优化。通过退城进园、搬迁技改，改造提升传统产业，己二酸己内酰胺一期、3万吨高性能浸胶帘子布等项目基本建成。通过开放招商、承接转移，加快培育新兴产业，平煤神马集团与中兴集团签订战略合作协议，中民投光伏发电、中兴科技产业园等项目开工建设，非煤产业、装备制造业比重分别提升5.1个和2.6个百分点。服务业发展计划深入实施，开展了电商与知名企业对接活动，第三产业比重提高1.9个百分点。载体建设持续加强。紧盯产业集聚区晋位升级计划，月度现场办公、季度观摩点评，实现集聚区与行政区域管理套合，完成投资587.4亿元，规模以上工业增加值增长15.5%。实施服务业“两区”重点项目31个，完成投资71.7亿元。创新能力持续提升。出台了实施创新驱动战略推动经济转型升级的意见，新增国家高新技术企业17家、国家重点实验室1家、省工程技术研究中心9家，平煤神马集团进入国家自主创新领军企业试点。生态环境持续改善。重点节能减排和蓝天、碧水、乡村清洁等工程深入实施，大气污染防治和白龟湖饮用水源地生态保护不断强化，秸秆禁烧工作顺利完成。林业生态建设有效提升，完成造林6.7万亩、森林抚育5.8万亩。

（三）全力抓统筹强引领，城乡一体协调发展。城乡一体化示范区建设全面提速，完成郑万铁路平顶山站及周边规划设计，平安大道西延、集中供热管网一期等有序推进。老城区改造提升步伐加快，鹰城广场北部提档升级等进展顺利，城中村和旧城改造主体完工173万平方米。获得国开行、农发行支持棚户区改造资金22.5亿元。县城和小城镇加快建设，综合承载能力持续提升。新农村建设稳步推进，新建民居1313套，新建改建农村公路497公里，解决33.5万人饮水安全，完成5万人脱贫，1镇3村入选省首批美丽宜居乡村，6个村落入选省第三批传统村落名录。

（四）全力打基础固根本，现代农业不断壮大。现代生态循环农业试验区建设进展顺利。新建高标准粮田14.6万亩，新增农机总动力7.2万千瓦。新增市级龙头企业24家，培育2家省级产业化集群和1家省示范性产业集群，建成15个千亩以上都市生态农业示范园区，带动建设生态循环农田20万亩，顺义循环农业研究院挂牌成立。在全省率先启动生猪价格指数保险试点，与上海市农产品中心签订年销售生猪100万头、牛肉2万吨合作协议。农村土地承包经营权确权登记颁证试点有序推进，新增土地流转面积7.2万亩。宝丰成为全国土地抵押贷款试点县。完成北汝河等4条河道部分河段治理和澎河水库除险加固工程，非法采砂等涉河违法得到有效治理。

（五）全力促改革扩开放，动力活力持续增强。编制完成政府部门“三定规定”，制定58个市直部门的权力清单和责任清单，精简各类权限1078项。机关事业单位养老保险制度改革和工资制度改革有序推进，食品药品监管体制和党政机关公车改革基本完成。平顶山日报传媒集团挂牌成立。完成交通运输行政执法体制改革。中房公司、市自来水公司完成改制。出台推广运用政府与社会资本合作(PPP)模式指导意见，市污水处理厂PPP模式运作成功。宝丰、舞钢农商行挂牌成立。出台支持企业上市融资的指导意见，6家企业在新三板上市，50家进入新三板后备企业库。“三证合一、一照一码”制度全面实施，新登记各类市场主体增长29.5%。与中民投公司、国药集团、深圳海王集团、河南国控集团、河南盛润集团、珠海莱茵恒丰公司等企业签署了战略合作协议，引进了泰国SIC公司电子芯片研发生产等项目，实际到位省外资金增长8.1%。中德生态环保合作试点项目进展顺利，赴台文化经贸交流活动圆满成功。

（六）全力惠民生增民利，社会大局和谐稳定。十项重点民生工程全面完成。财政民生支出比重提高4.7个百分点。城镇新增就业9.8万人，失业人员再就业3.8万人，农村劳动力转移就业5.7万人。企业退休人员养老金人均月增212.3元，城乡居民养老金月人均提高到80元，城镇医保、城乡低保和农村五保标准得到提高。临时救助制度规范完善，舞钢市被列入全国“救急难”综合试点。新开工保障性住房142.1万平方米。第二期学前教育三年行动计划持续实施，启动职教攻坚二期工程。成功举办市第九届运动会。实施城市医院支援农村卫生工程，城乡居民健康档案建档率达97.5%。“查尽责、除隐患、保安全”活动扎实开展，安全生产专项整治和消防安全大排查大整治持续深化，打击和处置非法集资、社会治安综合治理、食品药品安全和信访维稳等工作不断加强，社会大局保持和谐稳定。

一年来，我们自觉接受人大监督、政协民主监督和社会监督，认真办理人大代表建议和政协提案，支持民主党派、工商联和无党派人士参政议政。加强法治宣传教育，全面推行行政执法责任制，深入推进服务型行政执法，深化政务公开，法治政府建设取得新进展。加强国防教育、国防后备力量及人民防空建设，驻平解放军、武警部队和民兵预备役人员为地方建设作出新贡献，双拥工作深入推进，军政军民团结更加巩固。民族宗教、审计、统计、史志、档案、盐业、地震、气象等工作都取得新成绩。

各位代表!

在严峻复杂的形势下，作为资源型城市，能够取得这样的成绩，实属不易。但我们也清醒认识到，经济社会发展仍存在一些突出矛盾和问题：受经济下行大势影响，生产总值、固定资产投资等没有完成预期目标。部分行业企业生产经营困难，一些企业亏损严重。产业结构依然偏重，接续替代产业支撑不足，自主创新能力不强。安全生产形势严峻，环境污染问题突出，非法集资等各类风险隐患增多，教育、医疗、养老等与人民期待还有较大差距。部分干部面对落后局面，缺少危机感、紧迫感、责任感，不会为、不想为、不敢为、乱作为问题依然存在。对此，我们一定高度重视、认真解决，努力让人民满意。

三、2016年重点工作

今年是“十三五”规划的开局之年，也是推进供给侧改革的攻坚之年。世界经济总体延续疲弱复苏态势；我国经济长期向好的基本面没有改变，但结构性矛盾突出，经济下行压力较大。虽然形势复杂严峻，但我市转型发展也面临不少机遇和有利条件，国内外产业转移仍在持续，改革开放红利加快释放，新的增长动力正在形成；经过多年转型，传统产业持续提升，新兴产业逐步崛起，发展载体更加完备，特别是随着郑万铁路、孟平铁路复线、沙河复航的建设，将融入郑州半小时经济圈，区位优势持续提升。我们既要正视问题、防范风险，做好应对困难局面的思想准备和工作准备，又要增强信心、奋发有为，打好供给侧结构性改革攻坚战。

主要预期目标是：生产总值增长7%以上，一般公共预算收入增长5%左右，固定资产投资增长13%左右，社会消费品零售总额增长12%，进出口总值增长7%，实际到位省外资金增长7%，城镇新增就业6万人以上，常住人口城镇化率和户籍人口城镇化率分别提高1.6个和1.4个百分点，城乡居民收入增长与经济增长基本同步，居民消费价格涨幅3%左右，节能减排、环境质量改善等约束性指标完成省下达任务，发展质量和效益明显提高，结构性改革取得实质性进展。

做好今年政府工作，要主动适应、积极引领经济发展新常态，突出去产能、去库存、去杠杆、降成本、补短板，着力提高供给体系质量和效率，转中求进、攻坚克难，更加注重稳增长、促改革、调结构、强基础、惠民生、防风险综合平衡，确保“十三五”良好开局。工作中要突出“一个主题”，坚持“六个必须”，就是突出转型发展这个主题，必须把思想启动作为转型发展的总开关，必须把开放带动作为转型发展的主战略，必须把创新驱动作为转型发展的新引擎，必须把深化改革作为转型发展的突破口，必须把项目建设作为转型发展的总抓手，必须把转变作风作为转型发展的有力保障，进一步厘清转型思路，完善转型举措，加大转型力度，加快转型速度，提升转型效果。

（一）推进结构性改革，提高转型发展质量和效益。在适度扩大总需求的同时，加强供给侧结构性改革，加大产业产品结构调整力度，为稳定增长、提质发展提供强大动力。

挖掘需求潜力。抓好第一批318个重点项目，抓紧开工己二酸己内酰胺二期、高新区电气装备产业园等项目，加快中兴科技产业园、中民投光伏发电等项目建设，尽快建成力帆树民摩托及电动车工业园、神马股份东厂区搬迁改造等项目，确保完成投资560亿元。加强项目谋划储备，争取更多项目纳入国家重大工程包。积极争取中央预算内资金、国家专项建设基金和政策性贷款。抓好舞钢市和石龙区独立工矿区改造、宝丰和鲁山县采煤沉陷区治理、湛南老工业区搬迁改造。促进新兴热点消费，加快发展以教育培训、医疗保健、家政服务为重点的服务消费，大力催生跨区跨境、线上线下、体验分享、电子商务、智能家居等信息消费，培育新能源汽车、电动车等绿色消费，持续提升以通信、家电、文化娱乐、家用轿车为重点的农村消费，落实好汽车下乡政策。

改造提升传统产业。综合运用退城进园、承接转移、技术改造、延链补链、兼并重组等手段，对传统产业进行“脱胎换骨式”改造。煤炭产业，大力调整煤炭产品结构，增加主焦煤和高品质电煤的生产，加大原煤洗选比重，实行精细化加工配送，积极发展氢、苯、焦油等深加工产品。化工产业，充分发挥资源优势和尼龙原材料基地优势，深度推进与国内外龙头企业的战略合作，积极吸引长三角、珠三角、闽东南等地客商来我市生产尼龙66、尼龙6下游产品，着力打造世界级尼龙化工产业园。装备制造产业，抓住实施中国制造2025战略的机遇，依托平煤神马集团、平高集团、平煤机、中材环保、隆鑫机车等骨干企业，进一步提高高压电器、矿山机械、电收尘器、工程机械等研发制造水平，积极发展高压直流电器产业，加快实施走出去战略，扩大市场占有率。推进信息化与工业化深度融合，大力发展智能制造、绿色制造。钢铁产业，加快技术创新和装备升级，发展特种钢板、高端不锈钢制品等产品，提高产品附加值，使其焕发新的活力和生机。建材产业，加快产品升级和结构调整，按照市场需求，开发新产品、拓展新市场，实现绿色、低碳、创新发展。轻工食品产业，完善服装、家用纺织品和高档面料产业链，提高酿酒、粮油加工、肉制品、陶瓷等产品加工水平，做大做强特色产业规模。

培育壮大战略性新兴产业。重点培育新能源、新材料、电子信息、医药和医疗器械、汽车及零部件等战略性新兴产业，打造平顶山经济升级版。新能源产业，抓住国家推动绿色发展的机遇，大力发展风能、太阳能、水能等可再生能源，加快装机总规模36.4万千瓦的3个风电场和3个光伏电站建设，新开工装机总规模64万千瓦的9个风电和光伏发电项目，推进鲁山花园沟抽水蓄能电站前期工作。依托平煤神马集团和平高集团，整合相关资源和企业，引进石墨烯、锂离子电池等关键技术和项目，谋划储能产业发展，争取建设储能示范基地，打造全省重要的新能源基地。新材料产业，加大关键技术研发，提高安全气囊丝、改性工程塑料、工业丝规模化生产水平，加大特种石墨、高性能碳化硅等材料应用推广，做好蓝宝石项目前期工作，打造全省重要的新材料生产基地。电子信息产业，发挥中兴科技产业园、清华紫荆科技园的带动作用，更多引进智能终端生产和配套企业，抓好尧谷光电、东陆高科、极智联合智能固网终端等重点项目，主动对接郑州航空港区智能终端产业，发展高端光电显示、半导体新材料等配套产业，打造全省重要的电子信息产业基地。医药及医疗器械产业，大力发展医疗器械、医用耗材、医疗保健、微创介入等高端产品，开展抗艾滋病毒新药临床试验等技术研究，加快培南类抗生素等新特药产业化步伐，扩大产业规模。汽车及零部件产业，推进与隆鑫集团、力帆集团、北汽福田集团、宇通集团的合作，积极承接整车制造和零部件总成能力转移，支持发展低速电动汽车，开发生产多用途、多品种新能源机车。

加快发展现代服务业。以服务业“62231”发展计划为抓手，推动生产性服务业向专业化和价值链高端延伸、生活性服务业向精细化和高品质转变。出台支持物流业加快发展的政策措施，加快苏宁物流园、圣光医药物流园、宝丰生态农果物联城等建设，推进物流园区智能化、信息化、标准化改造。加快金融业发展，积极引进银行、证券、保险等各类金融机构入驻我市或开展业务，完善现代金融产业体系，增强金融支持经济发展能力。实施旅游消费拓展、服务提升、提质增效工程，以尧山温泉旅游度假区为抓手，加强旅游基础设施建设，推进温泉、滑雪、水上娱乐等休闲度假旅游健康发展，提高现有景区管理水平，推动乡村旅游和旅游小镇建设。高度重视并积极发展电子商务，持续加强与知名电商企业战略合作，加快建设覆盖市、县、乡、村的电子商务应用体系和物流快递网络体系，力争叶县电子商务进农村示范县创建、城乡一体化示范区电商产业园建设取得新进展。抓住国家推动“健康中国”机遇，积极发展养老和健康产业。放宽市场准入，推动教育、医疗、体育等服务业加快发展。

促进房地产业健康发展。加大房地产去库存力度，出台棚户区货币化安置指导意见，提高棚户区改造货币化安置率；建立租购并举的住房制度，培育发展住房租赁市场；完善农民进城购房补贴等政策，支持缴存职工用公积金贷款改善住房条件，将有稳定劳动关系的人口纳入公积金覆盖范围。落实商品房项目配套设施建设计划。调整开发结构，加大中高端改善性住房供给力度。防控商业地产风险，鼓励房地产企业跨界发展。

强化企业服务。深化企业大服务专项行动，加强工业用地收储和银企、产销、用工、产学研对接。坚持分类指导，对产品有市场、有效益暂时遇到困难的企业，继续给以支持；对不符合能耗、环保、质量、安全等标准的企业，推动升级改造；对长期亏损、资不抵债、停产半停产的“僵尸企业”，逐步实现市场出清。落实国家减轻企业负担的政策措施，鼓励企业加强内部管理，实现降本增效。严厉打击强买强卖、强装强卸、封门堵路等行为，着力优化经济发展环境。

（二）加快新型城镇化，促进城乡区域协调发展。全面提高城镇化发展质量，拓展城镇化发展空间。完善城市规划体系。尽快完成郑万铁路平顶山站周边区域控制性详细规划，编制白龟湖环湖规划，开展轻轨沿线两侧用地控制性详细规划和城市设计。推进农业转移人口市民化。落实户籍制度改革和居住证制度，制定附着在户籍上的教育、医疗、就业等领域的配套办法，促进农村人口向城镇转移。维护进城落户农民土地承包权、宅基地使用权、集体收益分配权，消除农民进城的后顾之忧。增强城市承载能力。按照“三区一中心”功能定位，大力推进城乡一体化示范区建设，抓好平宝大道、未来路西延、焦庄水厂等基础设施建设，全面完成湖滨景观带提档升级，加快城乡一体化示范区高中、市第一人民医院新院区和中医院新院区建设。改造提升老城区，推进河滨公园、鹰城广场北部提档升级、供水管网改扩建等工程，抓好过街天桥、停车场规划建设；实施湛北路、湛南路、南环路延伸工程，实现东西贯通；推进稻香路延伸、诚朴路南延、东环路南延、湛河东段3座大桥等项目建设，打通“断头路、丁字路”。加快湛南新城建设和新老城区公共交通对接。加强公共消防设施、消防队站、消防装备等设施建设。深入推进城市执法体制改革，强化城市管理数字化平台建设和功能整合。加强县城和小城镇建设。提升县级城市管理水平，完善产业功能、服务功能和居住功能，培育壮大特色产业集群，提高县域经济发展水平，增强辐射带动乡村发展的能力。加强乡镇政府所在地基础设施和公共服务设施建设，每个县（市）选定1-2个中心镇，强化规划指导和政策资金倾斜。支持舞钢市开展新型城镇化试点。抓好新农村建设。按照“五规合一”要求，完善推进新农村规划建设。继续实施农村公路建设三年行动计划，新建改建农村公路460公里。实施农村饮水安全巩固提升工程。持续开展农村人居环境综合整治，加强农村环卫设施建设与管护，探索建立村庄保洁长效机制，建设美丽宜居乡村。

（三）发展现代农业，推动农业提质增效。落实粮食安全市长县长责任制，划定永久基本农田并实行特殊保护，推进高标准粮田“百千万”工程，新建高标准粮田13万亩，确保粮食总产稳定在150万吨以上。推进现代生态循环农业试验区建设，启动平顶山食品加工园区规划建设。发挥宝丰顺义、舞钢瑞祥、红英农业等龙头企业的示范带动作用，新建改建生态循环规模企业20家，努力打造20万亩绿色（有机）粮食基地、40万亩饲料粮基地、全国韭菜种植示范基地、郏县油用牡丹农牧一体化基地。加强现代农业金融服务体系建设，支持市现代养殖合作总社发展壮大，提高综合授信能力。深入推进现代农业集群培育工程和都市生态农业发展工程，支持有条件的地方发展乡村特色产业集群和特色经济园区，新增现代农业产业化集群3家、农民合作社300家、家庭农场100家。加大农业供给侧改革力度，增加紧缺农产品生产，加强流通设施和市场建设，积极发展“互联网+现代农业”，提升同上海市农产品中心合作水平，提高农产品供给质量、效益和安全水平。落实农业“三项补贴”改革，完成农村土地承包经营权确权登记颁证工作，加快农村土地流转网络平台建设，力争实现县、乡土地流转服务体系全覆盖。加强现代农业研发基地建设，提升农业科研水平。完善农产品质量检测和追溯体系，培育知名品牌2-3家，认定鹰城名优品牌农业企业3家。

（四）加强基础支撑能力建设，厚植转型发展优势。统筹推进打基础管长远的大事、要事，加快推动单一优势、局部优势向综合优势转变。强化载体建设。突出集群、创新、智慧、绿色发展方向，完善协调推进机制，进一步优化规划布局，培育壮大产业集群，健全公共服务平台，强化基础设施建设，推动产业集聚区提质转型创新发展。年度完成投资600亿元左右，规模以上工业主营业务收入1200亿元，新增从业人员1.5万人；力争宝丰产业集聚区晋级一星，4家产业集聚区保持一星。加大服务业“两区”建设力度，开工建设市商务中心区鹰城中心、万达广场等项目，加快义乌商贸城、中原玉石城、叶县亿联建材家居五金城、宝丰万洋国际商贸城、石龙区温州商业街等项目建设，完成投资53亿元。增强重大基础设施支撑能力。大力推进郑万铁路平顶山段建设，全线开工沙河复航平顶山段工程，确保许南路等项目竣工，尽快完成大西环、平叶快速通道、平鲁快速通道前期工作，做好郑西高速鲁山段、平顶山机场、城市轻轨前期工作，新改建干线公路120公里。加强城市电网、天然气和热力管网建设，加快推进姚孟电厂2×100万千瓦机组、农村电网改造升级等项目。持续抓好北汝河综合治理、病险水库除险加固等工程建设，逐步建成现代水文水资源监测信息系统。深入实施“宽带平顶山”工程，加快4G网络、“光网城市”、宽带进乡入村、公共无线局域网建设。推进智慧城市、云计算中心建设，建立完善信息基础数据库和公共信息服务平台。加快电信网、广播电视网和互联网“三网融合”。强化人力资源支撑。实施人才强市战略，围绕重点产业和领域，突出“高精尖缺”导向，制定更加开放优惠的人才引进政策，开辟绿色通道，吸引国内外高层次人才。持续实施全民技能振兴工程，完善技能培训与产业发展对接机制，加强技师、高级技师培养，全面提高劳动者技能素质。加强经营管理人才引进和素质提升，建设高素质企业家队伍。健全人才服务保障体系，创造人才脱颖而出的良好环境。

（五）深化改革创新，增强转型发展内生动力。把改革创新贯穿于经济社会发展的各个领域，着力破除体制机制障碍，激发市场活力和社会创造力。加大重点领域改革力度。进一步简政放权，做好重点项目模拟审批试点工作，推进投资项目在线审批监管平台建设。巩固和扩大“三证合一”改革成果，强化事中事后监管。健全完善国有企业法人治理结构，继续剥离国有企业办社会职能和解决历史遗留问题，大力支持平煤神马集团、平高集团、舞钢公司、中材环保等企业发展混合所有制经济。全面清理限制非公有制经济发展的不合理规定，保护各种所有制经济产权和合法权益。加快建立现代预算制度，规范政府债务管理，推进财政管理流程化改革。依法加强财税征管，力争全面完成营改增任务，推进消费税改革和资源税费改革。加强不动产统一登记制度改革。加快农商行组建。加大企业上市培育力度。推广政府和社会资本合作模式。强化创新驱动。大力推进开放式创新，通过开放招商引进一批创新型企业、创新型团队和创新型项目，鼓励有条件的企业走出去，通过兼并重组、合资合作等方式建立研发平台。增强自主创新能力，强化企业创新主体地位，推动平高集团等大企业建设高水平研发中心，新建省级以上研发中心5家，争取在尼龙化工、装备制造、清洁生产等10大领域取得突破。支持企业与科研院所、高等院校深度合作，在人才技术密集城市引进或共建创新研发平台。鼓励支持大众创业、万众创新，推动“互联网+”与各领域深度融合、创新发展，培育新业态新模式新产业。整合产业发展转型资金用于支持科技创新，增加科学技术研究与应用资金。设立市科技创新风险投资基金，支持高科技企业发展。加快技术市场交易平台建设，提高科技成果转化率。

（六）强化开放带动，拓展转型发展新空间。落实对外开放“一把手”工程，坚持引进来与走出去双向开放，发展更高层次的开放型经济。积极融入国家“一带一路”开放新格局。推动能源、钢铁、化工、建材等企业到境外开展合作，开拓国际市场。支持平煤神马集团、平高集团等企业走出去，开展工程总承包。鼓励企业利用当前资产资源价格低的机遇，开展跨境并购。突出抓好开放招商。坚持大员上前线，大力实施以商招商、亲情招商、集群招商、园区招商和落地招商。通过开放招商，推进传统产业改造升级、战略性新兴产业培育壮大、服务业和现代农业加快发展。发挥好产业协会和异地商会作用，积极参加国家和省重大招商活动，办好第六届“华合论坛”。继续开展招商引资专项督察，提高合同履约率、资金到位率、项目开工率。加快出口基地建设。立足扩大出口、做强品牌、提高附加值，推动以平高集团为依托的电器装备、以舞钢公司为依托的特种钢材、以隆鑫公司为依托的摩托机车、以鲁山奔宝皮具为依托的小五金、以舞钢银龙和平棉公司为依托的纺织服装等出口基地建设，着力打造一批产业链完整、带动力强的出口产业集群。持续优化开放环境。完善财政支持外贸发展机制。积极推进海关和检验检疫机构建设。健全招商引资信息服务平台功能和外商投诉权益保护机制，打造规范有序、公平竞争的营商环境。

（七）保护生态环境，建设美丽鹰城。持续实施蓝天、碧水、乡村清洁等工程，坚持治、管、防、建一体抓，努力建设天蓝、地绿、水净的新鹰城。强化环境治理。持续改善大气质量，抓好工业大气污染防治、机动车污染控制、扬尘综合治理、秸秆禁烧及综合利用、燃煤污染防治、矿区环境综合整治等工作，实施工业污染源全面达标排放计划，完成电力企业火电机组超低排放改造，继续淘汰黄标车和老旧车，推进煤炭清洁高效利用和散煤替代。持续推进水污染治理，加快湛河综合治理工程，加强白龟湖上游入湖河流绿化治理及南水北调沿线污染企业排查，推进产业集聚区污水处理厂和城镇新污水处理厂及其配套管网建设，力争地表水出境断面达标。加强种植、畜禽养殖等农业面源污染防治，加快生活垃圾焚烧电厂建设，推进土壤污染治理及修复。开展环保综合督察和专项执法检查，严厉打击环境违法行为。加强资源管控。强化重点耗能行业和用能单位节能管理，推进建筑能效提升和绿色建筑产业链发展，实施节能节水行动计划，着力建设海绵城市。持续推进中德生态环保合作试点项目，支持中材环保等企业为绿色发展作出贡献。坚持节约集约用地，严格执行工业用地投资和产出强度标准。实行公共交通优先，加强公交场站建设，引导生活方式和消费模式向绿色低碳、文明健康转变。狠抓源头预防。继续实施林业生态建设提升工程，抓好生态廊道网络、农田防护林、城镇社区绿化美化等工程，完成造林21.8万亩、中幼林抚育5万亩。推进市北部山区生态建设、山顶公园周边区域环境治理和沙河治理工程，打造南水北调生态走廊。完善长效机制。健全领导干部生态环境损害责任追究机制，落实排污单位主体责任和行业管理部门监管责任。完善生态环境突发性灾害应急机制。推进环保机构监测监察执法管理体制改革，建立健全生态文明绩效评价考核和责任追究制度。

（八）建设文明鹰城，提升转型发展软实力。充分挖掘文化资源，加快文化改革创新，推动物质文明和精神文明协调发展。加强精神文明建设。积极践行社会主义核心价值观，推进群众性精神文明创建，加大全国文明城市创建力度。完善社会诚信体系建设，弘扬“开放、包容、务实、创新”的平顶山精神，倡导做文明人、办文明事，树立新时期平顶山人新形象。继续开展文化惠民工程，深入推进公共文化服务体系示范区创建。加强文化遗产保护，完成宝丰清凉寺汝官窑遗址保护展示工程。强化网上思想文化阵地建设，打造清朗网络空间。创新发展文化产业。推进大香山文化产业园、马街书会民俗文化园等建设。大力发展数字传媒、演艺业、工艺美术等特色产业，做大汝瓷、钧瓷、花瓷等产业规模。推进文化与旅游、科技、互联网融合发展，传统媒体与新兴媒体融合互动。深化文化体制改革。支持组建广播电视传媒集团。加快国有经营性文化单位改制，深化公共文化服务单位内部管理机制改革。

（九）保障改善民生，构建和谐社会。持续实施十项重点民生工程，让人民群众有更多获得感。打好脱贫攻坚战。贯彻精准扶贫、精准脱贫基本方略，加大财政金融扶贫力度，创新脱贫攻坚联动机制、扶贫资金整合机制，引导资金、土地、人才、技术、管理向贫困地区集聚，确保5万人脱贫。促进就业创业。完善创业扶持政策，加快建设一批创业孵化园区，以创业带动就业。强化对高校毕业生、农村转移劳动力、城镇困难人员等群体的就业援助，落实各项援企稳岗措施。做好退役军人安置工作。提高社会保障水平。落实企业最低工资制度，多渠道提高居民特别是中低收入群体收入。全面实施全民参保登记计划。进一步提高城乡低保补助水平，探索建立重特大疾病医疗救助制度，适当提高大病保险人均筹资水平和支付比例。规范完善企业养老保险和城镇职工基本医疗保险运行机制，深化机关事业单位养老保险制度改革，落实好县以下机关建立公务员职务与职级并行制度和乡镇工作补贴。探索政府购买服务等养老模式，力争社区老年人日间照料中心全覆盖。加快市儿童福利院和未成年人救助保护中心项目进度。推进保障性安居工程建设。鼓励发展慈善事业。建立困难残疾人生活补贴和重度残疾人护理补贴制度，健全农村“三留守”人员关爱服务体系。全面发展社会事业。持续实施第二期学前教育三年行动计划，扩充城镇义务教育资源，推进教育园区规划建设，办好特殊教育，推动平顶山学院转型发展。加快市第二人民医院新病房楼、儿童医院、精神卫生中心、传染病医院新病房楼等项目进度，加强基层卫生服务能力建设和疾病预防控制。落实“两孩”政策，改革完善计划生育服务管理。做好市科技馆、老干部活动中心前期工作，争取早日开工。争创全国双拥模范城“五连冠”。进一步加强社区建设。启动建设市体育运动学校综合训练馆，创建省全民健身示范城市。切实维护社会稳定。持续做好打击和处置非法集资工作，最大限度减少群众损失。严格落实安全生产责任制，抓好重点行业领域专项整治，消除重大安全隐患，坚决遏制重特大事故发生。加强社会治安综合治理和应急指挥系统建设，做好食品药品安全、信访稳定等工作，确保社会大局和谐稳定。

加强新形势下国防教育和国防后备力量建设，支持国防军队改革及驻平解放军、武警部队、预备役部队、民兵和人民防空建设。发挥工会、共青团、妇联等人民团体桥梁纽带作用。促进民族团结进步、宗教关系和谐。做好第三次全国农业普查工作。继续做好史志、档案、盐业、地震、气象等工作。

（十）抓好政府自身建设，打造法治高效廉洁政府。提升治理水平和能力，增强政府执行力和公信力。转变政府职能。深化行政审批制度改革，进一步清理审批事项、审批中介服务，全面建立权力清单、责任清单和负面清单制度，逐步实现“互联网+政务服务”，不断提高行政效能。强化市场监管、公共服务、社会治理等职能，推进公共服务提供主体和提供方式多元化。推进依法行政。严格执行人大及其常委会的决议决定，自觉接受法律监督和工作监督，主动接受政协民主监督，认真办理人大代表建议和政协提案，广泛听取民主党派、工商联、无党派人士意见，自觉接受舆论监督和社会监督。更加注重运用法治思维和法治方式解决问题，加强公务员法律法规培训。全面推行政府法律顾问制度，健全公众参与、专家论证、风险评估、合法性审查、集体讨论决定等重大行政决策程序。持续推进服务型行政执法建设。启动政府立法工作。深化政务公开，让企业和群众办事更加便捷。改进工作作风。践行“三严三实”要求，推动作风建设常态化。强化责任意识、担当意识，坚持深入基层、深入群众，问计于民、问政于民，用心想事、尽心干事，以严和实的作风破解难题。建立完善大督察机制，强力推进懒政怠政、为官不为问题专项治理，切实解决不会为、不想为、不敢为、乱作为等问题。旗帜鲜明地鼓励担当者、保护改革者、宽容失误者，使敢抓敢管、善做善成的干部得到表彰和重用，营造风清气正、干事创业的良好氛围。加强廉政建设。严格落实党风廉政建设责任制和主体责任，认真履行“一岗双责”，把廉洁从政贯穿到政府工作各个环节。完善惩治和预防腐败体系，严肃查处各类违纪违法行为，加强对重点领域、重点部门、重点项目的行政监察和审计监督，保持惩治腐败的高压态势。以反腐倡廉的成效，塑造良好的政府形象，凝聚转型发展的合力。

各位代表!

梦想唤起激情，奋斗铸就辉煌。让我们更加紧密地团结在以习近平同志为总书记的党中央周围，在省委、省政府和市委的坚强领导下，万众一心、锐意进取，为实现“十三五”良好开局、加快建设资源型城市可持续发展示范区、全面建成小康社会而不懈努力!
